# Unveiling the geography of historical patents in the United States from 1836 to 1975

**DOI:** 10.1038/sdata.2016.74

**Published:** 2016-08-30

**Authors:** Sergio Petralia, Pierre-Alexandre Balland, David L. Rigby

**Affiliations:** 1 Faculty of Geosciences, URU, Utrecht University, Utrecht 3584CS, The Netherlands; 2 CIRCLE, Lund University, P.O. Box 117S-22110, Lund, Sweden; 3 Department of Geography, University of California, Los Angeles, California 90095, USA

**Keywords:** Government, Geography, Economics

## Abstract

It is clear that technology is a key driver of economic growth. Much less clear is where new technologies are produced and how the geography of U.S. invention has changed over the last two hundred years. Patent data report the geography, history, and technological characteristics of invention. However, those data have only recently become available in digital form and at the present time there exists no comprehensive dataset on the geography of knowledge production in the United States prior to 1975. The database presented in this paper unveils the geography of historical patents granted by the United States Patent and Trademark Office (USPTO) from 1836 to 1975. This historical dataset, HistPat, is constructed using digitalized records of original patent documents that are publicly available. We describe a methodological procedure that allows recovery of geographical information on patents from the digital records. HistPat can be used in different disciplines ranging from geography, economics, history, network science, and science and technology studies. Additionally, it is easily merged with post-1975 USPTO digital patent data to extend it until today.

## Background & Summary

The long-run development of societies depends on the rate at which they innovate. Innovation not only defines opportunities for economic progress but also determines the way that knowledge itself is produced. Invention is increasingly collaborative, generated overwhelmingly within the dense agglomerations of individuals and firms that comprise the world’s major urban areas. Innovative cities are at the top of the global value chain, they are characterized by relatively high income per capita and by continuous improvements in average living standards. Clearly, not all residents of the largest cities benefit in the same way from invention, just as not all cities, regions and nations are as inventive as others. At the broadest scales, differences in rates of knowledge production over space and time are linked to geographical factors^1^ and to institutions that shape the character of economic and political fortunes^2^. Still, we know relatively little about why particular technologies were developed in some places rather than others, about why specific cities boomed on the backs of some ideas, while other places with competing innovations languished. How do chains of technologies emerge over time building industries and regions in different places while destroying older regimes? And, in a new era of rapid information flow, are the old canons of uneven historical development likely to be discarded or merely revised?

**Figure 1:**
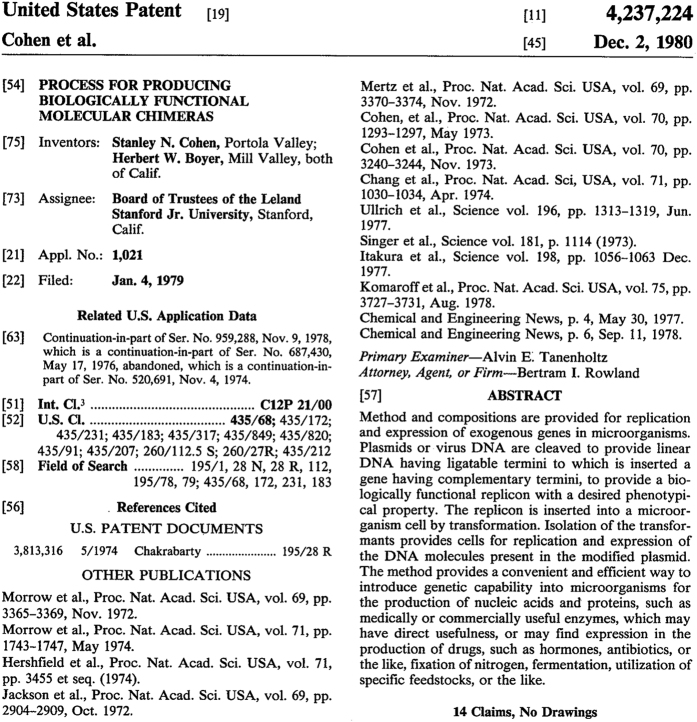
Original image of the front page of the Cohen-Boyer rDNA patent granted by the USPTO in 1980. The front page shows the different types of systematic information that a patent document contains, such as the inventors’ home addresses, the technological fields, and the references to prior art.

At this time, few options exist for scholars seeking to analyse historical data linking the types of technologies invented to their place of invention. The primary source of information on the geography of knowledge production is the patent document. A patent provides exclusive intellectual property rights on an invention to its inventor (or assignee). In this way patents encourage the development of ideas. More precisely, the USPTO defines a patent as, ‘…the right to exclude others from making, using, offering for sale, selling or importing the invention’. In exchange for such rights, the inventor (or assignee) is requested to provide detailed public disclosure of the patented invention. Public disclosure was designed to spur the diffusion of new ideas. Disclosure has also been key for academic researchers, providing a wealth of information on the business of science. By way of example, Fig. 1 shows the first page of the Cohen-Boyer rDNA patent that gave birth to the biotechnology industry. Like all patents, this document contains systematic information about the invention, the grant date, the name of the inventor(s) and their home address(es), the name of the assignee and its business address, the date of application, the technological domains to which the patent applies, reference to prior academic publications and other patent documents on which the invention builds, and a brief abstract of the invention. This information is regularly used in economics^3^, geography^4^, and science and technology studies^5^.

Although patent data are freely available from the USPTO Patent Full-Text and Image Database, they are not always available in a format that can be directly used for applied research. For some research questions, the raw data first have to be cleaned and processed (location disambiguation, or inventor/assignee name disambiguation for instance). A few structured, geo-referenced datasets have been developed over the past couple of years. One of the most commonly used is the patent dataset of the National Bureau of Economic Research (NBER), providing information on the state of first inventor for patents from 1975 to 1999. Another widely used database for US patents is the Patent Network Dataverse^6^, providing longitude and latitude coordinates of inventor addresses for patents granted by the USPTO from 1975 to 2010. In a similar fashion, the REGPAT dataset of the Organisation for Economic Co-operation and Development (OECD) provides inventor locations (NUTS3 level for Europe, TL3 for other OECD countries) for patents filed to the European Patent Office (EPO) or to the World Intellectual Property Organization (WIPO) from 1978 to 2011 (OECD, 2015).

However, these datasets only provide detailed geographical information on patents granted since 1975, the year when the USPTO began to record patents electronically. The main objective of this paper is to present HistPat, a well-structured, ready-to-use, comprehensive, and geo-referenced dataset of historical patents in the United States covering the years 1836 to 1975. HistPat contains geographical information (at the county level) on approximately 2.8 million patent documents (around 83% of all patents granted to US residents).

**Figure 2:**
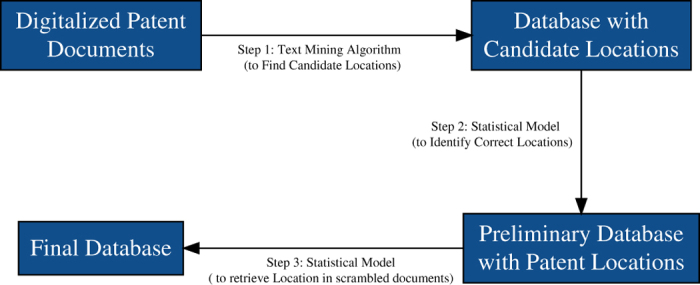
Data collection workflow in three main steps: (1) find potential locations within patent documents, (2) identify correct locations from all possible candidate locations, and (3) retrieve the geographical location in scrambled patent documents.

HistPat is built using optically recognized patent documents made available by Reed Tech and Google. We develop a methodological procedure to retrieve geographical information from those patent documents that can be divided into three steps (see Fig. 2 below). First, we use a standard text-mining algorithm to find potential locations within these patent documents. Second, we propose and calibrate a statistical model to identify correct locations from all possible candidate locations. Third, we exploit data from related patents to geolocate scrambled documents. HistPat is a valuable database that should be of interest to researchers in disciplines such as geography, economics, history, network science, and science and technology studies.

## Methods

This section describes the methodological procedure used to obtain the location of inventors and/or assignees from optically recognized patent documents (plain text documents). It is divided into three steps, as described in Fig. 2.

### Step 1: Finding candidate locations within patent documents

The final database on the geography of historical U.S. patents—HistPat—was built using bulk data from the United States Patent and Trademark Office. In 2006, the USPTO entered into a series of agreements with Reed Tech and Google to digitalize all available patent documents, making historical patent data available in bulk form. This bulk data contains ZIP or TAR files with TIFF or PDF images, concatenated XML or structured ASCII files, and can be accessed at: http://www.uspto.gov/learning-and-resources/electronic-bulk-data-products. The dataset presented in this paper has been constructed using these data, covering a period ranging from 1836 to 1975. Even though the first patent dates back to 1790, coverage between 1790 and 1836 is scattered and not entirely reliable. This is because a fire at the USPTO destroyed the records of thousands of granted patents and pending applications in 1836. Individual patents can also be accessed without using bulk data through the ‘Google Patents’ search engine: https://patents.google.com.

In this subsection we outline a procedure to create a database of ‘candidate’ geographical locations from the digitalized patent documents. This database will later be evaluated to assess the likelihood that a ‘candidate’ location is the actual location of an inventor or an assignee. This procedure is divided into two stages. We first identify all possible candidate locations within patent documents. Second, we generate a set of variables providing information about those locations such as their proximity to inventors’ names, their position within the patent document, and other features.

Identification of candidate locations depends upon access to a comprehensive list of town, city, and county names within the United States. We use two sources for this task. The first is provided by the U.S. Census Bureau at https://www.census.gov/geo/reference/codes/place.html. The second is the online gazetteer provided by Falling Rain Genomics, Inc and available at http://www.fallingrain.com/world/US/. The gazetteer is used to supplement neighbourhood names that are sometimes missing in census data. Historical US patent documents reference the addresses of inventors and assignees by naming the town, county and state where individuals and/or firms were located. Armed with a list of place names within the U.S., standard text detection algorithms can be used to detect the presence of these names within patent documents. Fast and reliable packages for text mining algorithms can be found in R software. We use the stringr (version 1.0.0), stringi (version 1.0.1), and tm (version 0.6.1) packages^7^.

**Figure 3:**
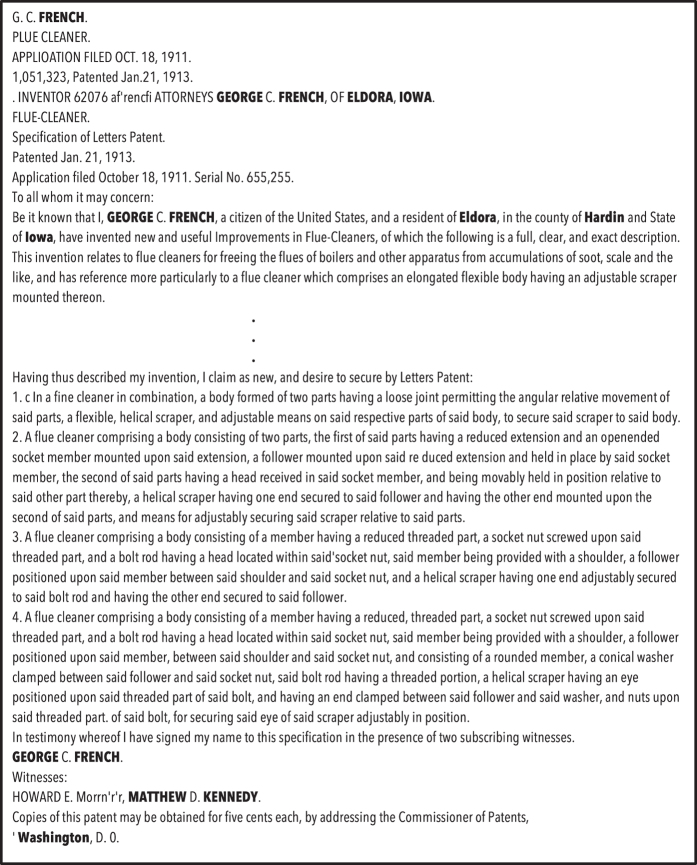
Example of an input in step 1. In this case, OCR text related to a patent on flue-cleaners by George C. French, a resident of Eldora, in the county of Hardin (Iowa).

Once we have a list of candidate locations we evaluate them in the context of the patent document, generating a set of covariates for each of them. Fig. 3 describes a typical input in this first step. Note that for any location name, an entire set of potential candidate locations may be generated, as there are many places with the same name in different states.

Table 1 (available online only) shows the typical output of this first step. The length of the document (in number of characters) is captured by the variable named ***‘Length’***. This variable remains constant within the document and it is best used in combination with other variables to standardize values and ease comparison across documents. The variable ***‘Location’*** identifies where in the document the name of the location was first found (as there may be multiple mentions of the same name). Additionally, other terms may be used (in combination with the location name) to create variables providing valuable information. For instance, the variable ***‘State’*** gives a value of 1 if the state name of the candidate location was found in the document. An additional variable measuring proximity between the name of the candidate location and the state name (if found), or inventor(s) name, also proved useful.

Constructing a set of variables for each potential location is crucial, as we will use them to evaluate the likelihood that each candidate location is the true location of the patent. Step 2 outlines a statistical procedure to filter out the correct locations from all available possibilities. Table 2 provides a detailed description of the variables we constructed.

### Step 2: Filtering correct locations

The objective of this subsection is to discuss the design of a statistical model that allocates probabilities to candidate locations that signify their likelihood of being the real location of a patent. These probabilities are generated by using the observed attributes of each location (see Table 2).

We do this by training and evaluating the predictive performance of three popular and well-studied statistical procedures (Neural Networks (NN), K-th Nearest Neighbours (KNN), and a Probit model). For training purposes we use a manually collected sample that identifies the correct locations for a randomly selected subset of patent documents. We input manually the correct location for approximately 7000 patent documents, which were selected randomly from all available patents covering the period 1836 to 1975.

More specifically, let the output or response variable of the statistical model take two possible values from the finite set *Y*={0,1}; where the category ‘1’ identifies correct locations within patents. Let $X=X_{ij}=(X_{ij}^{1},X_{ij}^{2},\ldots ,X_{ij}^{p})$ be a vector of *p* predictors (or attributes) for location *i* within patent document *j*. If we treat *Y* as a quantitative output we allow predictions of *Y* (denoted $\hat{Y}$) to fall within the interval [0,1]. Additionally, assume there exists a set of measurements (*x*_*ij*_, *y*_*ij*_) for a randomly selected subset of patents *j*=1,…,*N* that we will call the training set.

Statistical decision theory provides a framework to evaluate problems of this sort. Within this framework we aim at finding a function *f*(.) to predict *Y*_*ij*_ given *X*_*ij*_. This framework requires specifying a loss function *L* (*Y, f* (*X*)) that penalizes errors in prediction. We seek to find an approximation $\hat{f}\left(X\right)$ to the relationship between the predictors and the output *Y*. Probit and NN models can be grouped within the class of Projection Pursuit Regressions^8^ where *f*(*X*) can be defined as

$$f\left(X\right)=\sum_{m=1}^{M}g_{m}\left(\omega_{m}^{T}X\right)$$

with a loss function of the form

$$L\left(Y,g_{m}\left(.\right),{\;\omega }_{m}^{T},\;X\right)=\sum_{i=1}^{N}{\left[y_{i}-\sum_{m=1}^{M}g_{m}\left(\omega_{m}^{T}x_{i}\right)\right]}^{2}$$

Our aim is to approximate the parameters of this model by minimizing the loss function. What differentiates Probit and NN models are the assumptions over the parameters. If we let *M*=1 and assume *g*_*m*_=*g* to be the Cumulative Distribution Function (CDF) of the standard normal distribution we get a Probit model. What differentiates NN models is that they use linear combinations of the predictors to construct a set of indexes *Z*_*m*_ that are combined in linear form to estimate $\hat{Y}$ . Thus,

$$Z_{m}=\sigma \left(\alpha_{0m}+\alpha_{m}^{T}X_{m}\right),\;m=1,\ldots ,M$$

$$T_{}=\beta_{0}+\sum_{m=1}^{M}\beta_{m}^{T}Z_{m}$$

f(X)=g(T)

where the activation function *σ* and the output function *g* could be chosen to be the logistic function. Note that the NN model proposed here can be understood as logistic regression using *Z*_*m*_ as covariates. The intermediate inputs *Z*_*m*_ are called hidden units because their values are not observed directly.

The last statistical model we implement does not require any statistical fitting. The KNN model consists of finding for any given point *x*_*o*_, the K-th nearest neighbours within a set of training points (*X*,*Y*); to later classify *x*_*o*_ using a decision rule based on the information provided by the K-th nearest neighbours. We use the Minkowski distance metric to find the nearest neighbours. Predictors are standardized beforehand, we set *θ*=2.

$$Distance\left(x_{o},x_{ij}\right)={\left(\sum_{r=1}^{p}{\left|x_{o}-x_{ij}\right|}^{\theta }\right)}^{1/\theta }\;with\;\theta >1$$

After the K-th nearest neighbours are found, *x*_*o*_ is classified implementing a decision rule over all output values within the neighbourhood of *x*_*o*_ $\left(i.e.\;all\;y_{ij}\varepsilon \;{Ν}_{x_{0}}\right)$. We use the Epanechnikov kernel function to weight neighbours according to their distances and predict the value of *y*_*o*_ as a weighted average of all $y_{ij}\varepsilon \;{Ν}_{x_{0}}$.

Predictions of these procedures ($\hat{Y}$) will lie in in the interval [0,1]. We can then classify each location within the groups *G*={*Correct*, *Incorrect*} according to the following rule:

$$\hat{G}=Correct\;if\;\hat{Y}>µ$$

$$\hat{G}=Incorrect\;if\;\hat{Y}\leq µ$$

where μ is a threshold parameter that falls in the interval [0,1], used to discriminate correct from incorrect locations. This might be interpreted as a threshold likelihood that potential locations should pass to be considered as real locations in our database. This classification rule will typically be subject to misclassification error. However, as μ increases, the probability of misclassifying an incorrect location should decrease. Table 3 shows a typical output. Note that the table includes a new variable with the value of $\hat{Y}$ for each location. In this example we only keep the locations predicted as true with likelihood above 50%.

Note that the three statistical models proposed in this paper can be clearly ordered in terms of their parametric constraints. The Probit model, being the most restrictive of all, has the advantage of speed as the number of parameters to be learned from the data is lower. Commonly used searching algorithms, such as iteratively reweighted least squares (IRLS), can be used to choose the parameters that minimize the loss function. An additional feature is that we are able to provide an interpretation of the effect of our predictors on the output.

It is often the case, however, that NN and KNN models outperform Parametric Single Index Models (PSIM) in terms of predictive power^8,9^. As prediction is the main objective, the simplicity and interpretability of PSIM may impose constraints we don’t want or need. NN models are more flexible and have been proven to approximate nonlinear relationships relatively well. They tend to outperform PSIM in most empirical applications^8,9^. There are, however, some shortcomings. First of all, it is usually difficult to interpret the effect of predictors as they are masked within the hidden units. Additionally, NN models tend to have a considerable number of weights to be estimated, often leading to the risk of overfitting the data if parameters and optimization procedures are not chosen appropriately. We use the so-called resilient back-propagation algorithm to minimize the loss function^10–12^. This modifies weights after calculating the gradient of the error function until a local minimum is reached. An appealing feature of this procedure is that different learning rates can be assigned to different weights that make the procedure more robust when compared to traditional back-propagation algorithms.

Being completely non parametric, KNN models tend to impose an even higher computational burden. Note that they usually require finding the neighbours and storing the entire training set to be matched against query points. In our case it requires N*p operations per *x*_*o*_. However, KNN models have proven successful in a variety of classification problems, especially when decision boundaries are very irregular^8,13,14^. An appealing feature is that KNN models are unstructured and don’t impose any particular parametric restriction, nor do they require any model to be specified. As in the case of NN models, they are not useful for understanding the relationship between the predictors and the outcome and may be unstable under some circumstances.

As our final goal is to correctly predict as many locations as possible while minimizing errors, the final decision over competing alternatives will be entirely based on predictive performance. The inclusion of these three particular competing alternatives is based on the wide variety of scenarios they could accommodate. The idea is that other researchers wanting to expand or improve this database could have a set of flexible tools at their disposal. Note that for the particular problem at hand we have an important advantage over the usual predictive endeavours for we can see the Data Generating Process (DGP) via the patent documents themselves. Moreover, this DGP barely changes over time. This means that we can create attributes of locations knowing beforehand whether and how they will work.

By way of an example, let us say one is interested in tracking down the emergence of new technologies or chemical components by searching for references to those technologies (i.e., internal combustion, polyethylene, etc.). In principle, the same exact procedure could be applied to the set of available documents, replacing location names by these keywords. If these keywords appear in any part of the document, evaluation of the appropriateness of located terms may be more difficult. If decision boundaries are more irregular, less restrictive approaches may be preferable.

### Step 3: Including location for ‘unreadable’ patent documents.

At this stage we have a preliminary database with the correct locations (or predicted correct) for around 2.65 million patents documents. Even though the Optical Character Recognition (OCR) software succeeded in providing an accurate and detailed digitalized description of most patent documents, some of them still remained ‘unreadable’ (or ‘machine-unreadable’ to be fair). This means the OCR software was unable to recognize scrambled, broken and unconnected characters or symbols for some documents. As a result, locations could not be retrieved for those patents.

It is possible, however, to make use of the bibliographic information on patents to infer a location for those ‘unreadable’ documents. This can be done by evaluating other patents of the same inventors and/or assignees. The idea is to evaluate, for all ‘unreadable’ patent documents, a set of potential locations using the predicted locations in step 2 and the fact that we often have bibliographical information related to the patent. See for instance the following example of a scrambled document with an ‘unreadable’ location: https://www.google.com/patents/US6469. Available bibliographical information can be found here: http://worldwide.espacenet.com/publicationDetails/biblio?CC=US&NR=6469.

In this step, we create a set of ‘potential’ locations for every ‘unreadable’ patent whenever the same inventor or assignee has another patent with an identified location (retrieved in step 2). As in step 2, we create a set of attributes for ‘potential’ locations that will be related to the number of times that location was found in other patents under the same inventor/assignee name, the ubiquity of the inventor/assignee name, etc.

**Figure 4:**
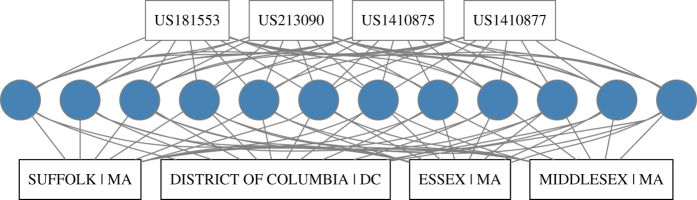
This figure describes how to find location for unreadable patent documents. We use here a set of patents invented by Alexander Graham Bell as an example. Patent numbers at the top of the figure correspond to documents for which a location couldn’t be found, while blue coloured dots represent patent documents with an identified location from step 2. The division between unreadable and readable patents is only for illustrative purposes. All of these patents contain an assigned location from step 2. Lines connecting unreadable patents with blue dots mean those patents share the same inventor name (i.e., Alexander Graham Bell). We can connect those blue dots with found locations to create a geo-referenced database of unreadable OCR text.

Figure 4 below summarizes this procedure. It describes how to construct a database of possible locations for ‘unreadable’ patent documents using the bibliographical information about the inventor/assignee name.

In this example there are multiple locations to consider because the inventor lived in different places. Table 4 shows how we structure the information displayed in Fig. 4. The variable named ***‘Frequency’*** counts how many times that location appeared. Note that this variable does not vary across patents, as it is a characteristic associated with the inventor name rather than the specific patent document. However, we can make use of the bibliographical information to include variables that capture the heterogeneity across ‘unreadable’ patents by identifying, for instance, how many of the blue-coloured patents were filled in the same year. The variable ***‘Year Coinc.’*** counts how many of the positive matches in ***‘Frequency’*** correspond to the same year of the ‘unreadable’ patent. This variable provides valuable information to disambiguate among locations when the inventor has moved during the period, as in this case (the correct location for patents US1410877, US1410875, and US213090 is Washington DC, and Boston MA for US181553). Information about technological classes may also help disambiguating among locations when the inventor name is very common, by considering also the area of expertise of the inventor.

Note that the information provided by the coincidence of class and year can be incorporated directly in the network of Fig. 4. It is possible to create additional networks that link ‘unreadable’ patent documents only with patents of the same inventor and within the same technological class. Frequencies can be calculated for this sub-network. This procedure has the advantage of reducing the dimensionality of the network, which may be handy when the number of pairs to evaluate is very high, as in this case.

We filter correct locations at this time in exactly the same way as in step 2 (above). We divide the manually collected sample into training and test sets to train the same three econometric procedures and evaluate them according to their predictive performance. Note that any errors already present in the preliminary database coming from step 2 may be carried to this stage. However, the threshold coefficient μ can be set arbitrarily to determine the desired error tolerance level. Locations predicted as ‘correct’ will be appended to the database of step 2. Table 5 lists variables we constructed for this step.

### Code availability

All procedures implemented in this project were written in R software (Version 3.3.1). We used text mining algorithms from the following packages: stringr (version 1.0.0), stringi (version 1.0.1), and tm (version 0.6.1). We provide a simplified example of the original code to facilitate the reproduction of the procedures described in this paper, with access details provided in the Data Citation 1, under the name ‘Replication Example’. The entire code is available upon request.

## Data Records

The result of this procedure is a database that we will refer to as HistPat. HistPat and supporting data are archived at the Harvard Dataverse, Harvard University, with access details provided in the Data Citation 1. The ‘HistPat.csv’ file, within the folder named ‘HistPat Dataset’, contains seven columns and 3,496,301 rows. Each row corresponds to a location in a patent document while the columns provide the following information:

**Variable Name: Description**

**PN:** Patent Document Publication Number as shown in patent documents

**FIPS:** County subdivision FIPS code as specified by the US Census Bureau (https://www.census.gov/geo/reference/codes/place.html)

**State:** State postal code as specified by the US Census Bureau (https://www.census.gov/geo/reference/codes/place.html)

**County:** County Name

**Source:** Identifies how the patent location was obtained. One of the following types:

- MCS: Manually Collected Sample
- MCU: Manually Contributed by Users
- Step 2: Automatically inputted, corresponds to the second step described in this document
- Step 3: Automatically inputted, corresponds to the third step described in this document

**Alpha:** Expected accuracy for automatically derived locations. A value of 5, 2.5, or 1 means that you should expect 5, 2.5, or 1 wrongly assigned locations every 100 patent documents, respectively

**Year:** Year of publication (grant year)

The variable ‘PN’ gives the patent publication number, as shown in patent documents. Users can search individual patents listed in HistPat by copying and pasting this patent document publication number in the Google Patent search engine: https://patents.google.com/patent/. For instance, the patent for the phonograph (PN=US200521), invented by Thomas Edison in 1878 can be found at this address: https://patents.google.com/patent/US200521.Users can also use this number to append HistPat to other existing datasets such as the NBER patent data^15^. In this case, the corresponding variable name is ‘patent’, and only includes numeric values, i.e., ‘US200521’ would be ‘200521’.

## Technical Validation

All three procedures have tuning parameters to be learned from the data or to be imposed exogenously. For instance, in the case of NN models, weights are learned from the data while the number of hidden units is usually set by the researcher. In Probit models coefficients are estimated, while the number of neighbours in KNN models is usually chosen exogenously. Results of this section are obtained using 25 neighbours for the KNN model, and allowing only one layer and 30 hidden units in the NN model. Results are robust to departures from these values.

In this section we test the performance of these three different alternatives. The main goal is to choose the best procedure in terms of predictive accuracy and coverage. A performance assessment over an independent test set is crucial for this sort of procedure as there is a risk that models will over-fit the training set. Over-fitting the training set occurs when parameters of the model are tuned in such a way that they become suitable only for that particular training set, without being able to generalize and correctly predict new data. In a first subsection (a) we provide a comparison across procedures using training and test samples of equal size. We use these results to choose the ‘best’ performing procedure.

After having chosen the ‘best’ performing model we also test whether predicted locations evidence any sort of bias. Note that we are identifying locations by name matching, based on an imperfect OCR procedure. It may be the case that some particular locations are either more difficult to recognize or to evaluate properly. For instance, as the length of the location name increases, the likelihood of misspelling increases too. However, if a location with a long name is detected, the likelihood the model considers it correct increases. This may generate a bias towards correctly predicting some locations more often than others. We test this in the second subsection (b) by comparing the distribution of locations in our final database to the one collected manually, both across time and technological domains.

In both subsections (a) and (b) we only show the result of evaluating all three procedures for what we called the second step. This means that we only include the comparison across procedures for the case where we aim at predicting which candidate locations are correct. Remember that we apply a similar procedure also to identify the locations in scrambled documents (i.e., step three). We do not show the comparison for this later case because predicting performance of models is almost identical to the one obtained in step 2.

### Predicting model performance

**Figure 5:**
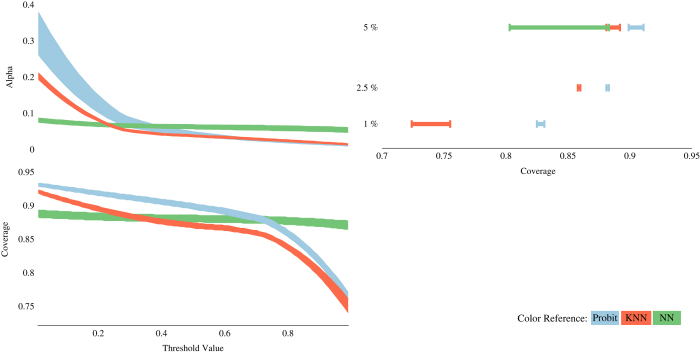
Predictive performance of the different models (Probit=blue; NN=green; KNN=red) in terms of coverage (share of geo-referenced patents) and reliability (probability that the location is correct).

Figure 5 compares all three alternatives in terms of their predictive performance. We use the value μ to filter locations that are predicted to be true above the specified threshold. We evaluate, for any value of μ, how many errors are contained in the final sample (we call this parameter alpha) and what percentage of all patents within the sample contain at least one location (we call it coverage). Specifically, we compute alpha (for a particular value of μ) as the percentage of locations that were wrongly codified as ‘correct’ (i.e., they passed the filter even though they are not the correct location of the patent). The final coverage is computed as the proportion of patents that have at least one location.

Figure 5 should be read in a clock-wise direction from the bottom-left. Different colours were assigned to alternative procedures (blue to Probit, green to NN, and red to KNN). The first graph shows how coverage decreases as we increase the value of our threshold (μ). As expected, requiring higher predicted probabilities decreases the coverage of the final sample. However, as can be seen in the second (top-left) graph, this also decreases the probability that we will commit mistakes and include ‘incorrect’ locations in the final sample. These two graphs share the horizontal axis and show how the coverage and alpha change as we move the threshold value μ. The thickness of the line represents the 95% confidence interval (calculated repeating 100 times the procedure for different randomly selected training and test sets). Note that the Probit model (blue) always has a higher rate of coverage than the KNN model (red). When it comes to avoiding mistakes, however, the Probit model is inaccurate for lower values of μ but quickly recovers and performs better than the KNN model for higher values of μ.

The NN model (green) seems to be very insensitive to changes in μ. In fact it starts being sensitive for values that are very close to 1, which cannot be captured by this figure unless scales are changed. This fact exemplifies the inappropriateness of using the threshold value μ as a reference to compare across models. Instead, we use μ to set up a level of alpha and then evaluate procedures by comparing their coverages. In this way we are able to fix the number of mistakes we are willing to commit, and then choose the preferred procedure as the one that maximizes the coverage.

The top-right graph ranks procedures (in terms of coverage) after having fixed a desired level of alpha (vertical axis). This graph shares the same vertical axis as the one on its left but it is scaled differently, only for three values of alpha (5, 2.5, and 1%). Note that the Probit model outperforms all other procedures as it obtains the highest coverage (horizontal axis) for any given value of alpha. This result also holds also when we compare procedures in step 3. As a result, we are going to predict whether candidate locations are correct relying on the probabilities we obtain after evaluating covariates of each location based on our calibrated Probit model.

### Geographical distribution of the final sample

One concern is whether the set of final locations in our sample (those that passed the Probit filtering) are representative of the true geographical distribution of patent locations. It may be the case that the ubiquity of some city names triggered false positives beyond what can be considered statistically acceptable. We propose to perform a Pearson’s chi-squared test to evaluate whether observed differences between the geographical distribution of the manually collected sample and our final database can be considered statistically insignificant. We use a final sample targeting an error rate (*α*) of 5%, meaning that we set the filtering parameter μ so as to admit, at most, a misclassification rate of 5%.

Let $p_{1}^{FD},p_{2}^{FD},p_{3}^{FD}\ldots p_{k}^{FD}$ be the proportion of patents coming from locations 1,2,3…*k* in the Final Database (FD) and let $p_{1}^{MCS},p_{2}^{MCS},p_{3}^{MCS}\ldots p_{k}^{MCS}$ be the proportion of patents in the Manually Collected Sample (MCS), for those same locations. Pearson’s chi squared test evaluates the following hypothesis:

$$H_{0}:p_{1}^{FD}=p_{1}^{MCS},p_{2}^{FD}=p_{2}^{MCS},\ldots ,p_{k}^{FD}=p_{k}^{MCS}$$

$$H_{1}:p_{i}^{FD}\neq p_{i}^{MCS}\;\mathrm{for}\;\mathrm{any}\;i$$

Note that the test will reject the null hypothesis if a significant difference is found for any particular location. The statistic is calculated as the sum of the standardized counts of all k locations, which is asymptotically chi-square distributed with k-1 degrees of freedom. Specifically, $X^{2}=N\sum_{i=1}^{k}p_{i}^{FD}{\left(\frac{p_{i}^{MCS}-p_{i}^{FD}}{p_{i}^{FD}}\right)}^{2}$, where N represents the total number of observations.

**Figure 6:**
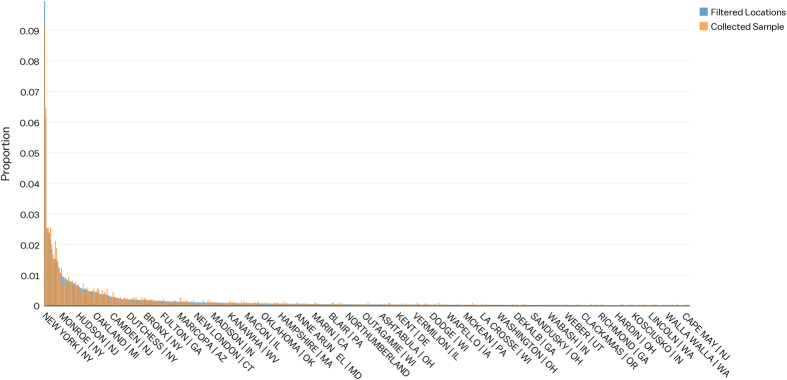
The graph compares the geographical distribution of the sample of patents collected by hand with the geographical distribution of the patents geo-referenced with our algorithm.

Figure 6 shows graphically how similar both samples are, in terms of their geographical distribution. In fact, the statistical test over these two distributions gives a value of $X^{2}=2705.5$ (with a p-value of 0.975) leading us not to reject the null hypothesis that both distributions are statistically equivalent.

We also test whether this result holds along the most relevant dimensions in our sample, by type of technological domain and over time. A genuine concern may be that the statistical procedure performs poorly for particular technologies; this may happen if some technologies use a vocabulary that makes detection harder. For instance, mining or extractive technologies may reference locations to describe soil characteristics increasing the likelihood the procedure will include a false positive. In addition, changes in the way documents have been constructed may also have an effect on the likelihood that correct locations are included.

**Figure 7:**
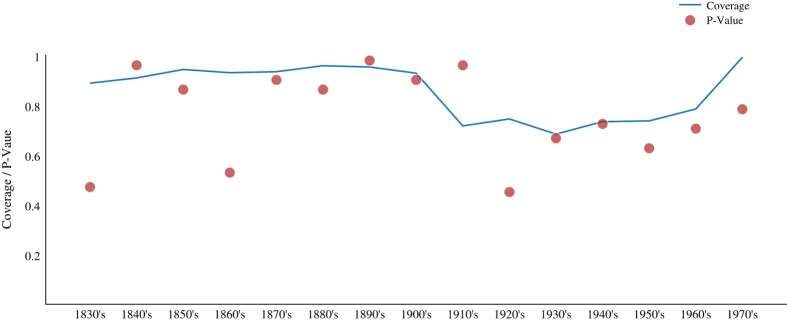
The graph shows the share of geo-referenced patents (coverage) for each period and the associated *P*-values. Red coloured dots represent the *P*-values of the statistical chi-squared test for each period, while the blue line shows the coverage of the sample over that period. As before, coverage is calculated as the proportion of patents with at least one location in the final database.

Figure 7 evaluates the similarity of the geographical distribution of the final database and the manually collected sample over different periods of time. We perform the same test as before but divide the sample into 15 periods of 10 years each.

Figure 7 shows that we maintain a relatively high rate of retrieval over all periods with the maximum value above 99% for the 1970s, and the lowest rates between 1910 and 1950 with a coverage that does not fall below 69%. Note that we never reject the null hypothesis that the final database is geographically unbiased for any period (the lowest p-value is 0.42).

**Figure 8:**
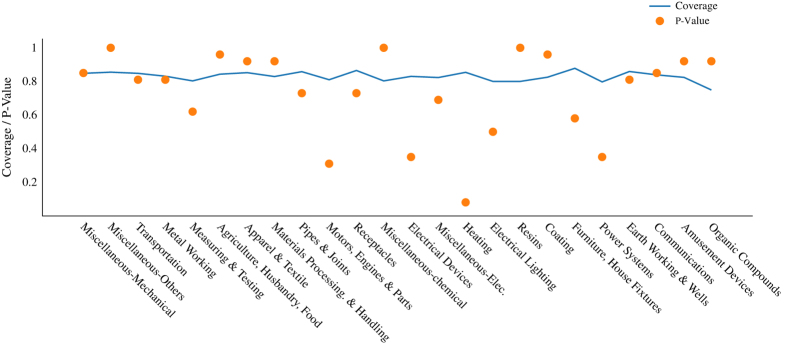
The graph shows the share of geo-referenced patents (coverage) for different technological fields.

Figure 8 below is analogous to the previous figure but considers different technological domains instead of time periods. As before, results show that we have a relatively high and homogeneous rate of coverage across technological domains. Also, we never reject the null hypothesis that our final sample is unbiased (the lowest p value is 0.15 for Heating technologies).

Recall that all these tests were done by setting a threshold level that corresponds to an error rate (alpha) of 5%. Even higher *P*-values are obtained at alpha values of 2.5 and 1%.

## Usage Notes

A more detailed visualization of the database (including maps) can be found at https://histpat.shinyapps.io/HistPat/. We plan to include new updates of this database to include manually collected data for those patents we could not retrieve automatically. We recommend checking for the latest version as we continuously update the database to include manually collected locations for those patents that couldn’t be input by one of our procedures.

## Additional Information

**How to cite this article:** Petralia, S. *et al.* Unveiling the geography of historical patents in the United States from 1836 to 1975. *Sci. Data* 3:160074 doi: 10.1038/sdata.2016.74 (2016).

## Supplementary Material



## Figures and Tables

**Table 1:** This table shows a typical output of step 1, in which the algorithm identifies potential candidate locations within patent documents

**Publication Number**	**City**	**State**	**Length**	**Location**	**State Located**
US1051323	ELDORA	CO	7482	150	0
US1051323	ELDORA	FL	7482	150	0
US1051323	ELDORA	IA	7482	150	1
US1051323	ELDORA	NJ	7482	150	0
US1051323	ELDORA	PA	7482	150	0
US1051323	ELDORA	WV	7482	150	0
US1051323	FEBRUARY	TN	7482	2211	0
US1051323	FRENCH	AR	7482	7	0
US1051323	FRENCH	ID	7482	7	0
US1051323	FRENCH	MN	7482	7	0
US1051323	FRENCH	NM	7482	7	0
US1051323	FRENCH	VA	7482	7	0
US1051323	GEORGE	IA	7482	129	1
US1051323	GEORGE	KS	7482	129	0
US1051323	GEORGE	MO	7482	129	0
US1051323	GEORGE	MS	7482	129	0
US1051323	GEORGE	MT	7482	129	0
US1051323	GEORGE	NC	7482	129	0
US1051323	GEORGE	OR	7482	129	0
US1051323	GEORGE	TX	7482	129	0
US1051323	GEORGE	WA	7482	129	1
US1051323	GEORGE	AR	7482	129	0
US1051323	HARDIN	AR	7482	426	0
US1051323	HARDIN	CA	7482	426	0
US1051323	HARDIN	CO	7482	426	0
US1051323	HARDIN	GA	7482	426	0
US1051323	HARDIN	IA	7482	426	1
US1051323	HARDIN	IL	7482	426	0
US1051323	HARDIN	KY	7482	426	0
US1051323	HARDIN	MO	7482	426	0
US1051323	HARDIN	MT	7482	426	0
US1051323	HARDIN	NC	7482	426	0
US1051323	HARDIN	OH	7482	426	0
US1051323	HARDIN	OR	7482	426	0
US1051323	HARDIN	TN	7482	426	0
US1051323	HARDIN	TX	7482	426	0
US1051323	IOWA	IA	7482	158	1
US1051323	IOWA	LA	7482	158	0
US1051323	IOWA	PA	7482	158	0
US1051323	IOWA	WI	7482	158	0
US1051323	KENNEDY	AL	7482	7355	0
US1051323	KENNEDY	CA	7482	7355	0
US1051323	KENNEDY	GA	7482	7355	0
US1051323	KENNEDY	IA	7482	7355	1
US1051323	KENNEDY	IL	7482	7355	0
US1051323	KENNEDY	IN	7482	7355	0
US1051323	KENNEDY	KY	7482	7355	0
US1051323	KENNEDY	MN	7482	7355	0
US1051323	KENNEDY	MO	7482	7355	0
US1051323	KENNEDY	NE	7482	7355	0
US1051323	KENNEDY	NM	7482	7355	0
US1051323	KENNEDY	NV	7482	7355	0
US1051323	KENNEDY	NY	7482	7355	0
US1051323	KENNEDY	PA	7482	7355	0
US1051323	KENNEDY	SD	7482	7355	0
US1051323	KENNEDY	WA	7482	7355	1
US1051323	KENNEDY	WI	7482	7355	0
US1051323	MATTHEW	KY	7482	7344	0
US1051323	MATTHEW	NC	7482	7344	0
US1051323	MATTHEW	TN	7482	7344	0
US1051323	MATTHEW	WA	7482	7344	1
US1051323	WASHINGTON	CT	7482	7466	0
US1051323	WASHINGTON	FL	7482	7466	0
US1051323	WASHINGTON	AL	7482	7466	0
US1051323	WASHINGTON	AR	7482	7466	0
US1051323	WASHINGTON	CA	7482	7466	0
US1051323	WASHINGTON	CO	7482	7466	0
US1051323	WASHINGTON	GA	7482	7466	0
US1051323	WASHINGTON	IA	7482	7466	1
US1051323	WASHINGTON	ID	7482	7466	0
US1051323	WASHINGTON	IL	7482	7466	0
US1051323	WASHINGTON	IN	7482	7466	0
US1051323	WASHINGTON	KS	7482	7466	0
US1051323	WASHINGTON	KY	7482	7466	0
US1051323	WASHINGTON	LA	7482	7466	0
US1051323	WASHINGTON	MA	7482	7466	0
US1051323	WASHINGTON	MD	7482	7466	0
US1051323	WASHINGTON	ME	7482	7466	0
US1051323	WASHINGTON	MN	7482	7466	0
US1051323	WASHINGTON	MO	7482	7466	0
US1051323	WASHINGTON	MS	7482	7466	0
US1051323	WASHINGTON	MT	7482	7466	0
US1051323	WASHINGTON	NC	7482	7466	0
US1051323	WASHINGTON	NE	7482	7466	0
US1051323	WASHINGTON	NH	7482	7466	0
US1051323	WASHINGTON	NV	7482	7466	0
US1051323	WASHINGTON	NY	7482	7466	0
US1051323	WASHINGTON	OH	7482	7466	0
US1051323	WASHINGTON	OK	7482	7466	0
US1051323	WASHINGTON	OR	7482	7466	0
US1051323	WASHINGTON	RI	7482	7466	0
US1051323	WASHINGTON	SC	7482	7466	0
US1051323	WASHINGTON	TN	7482	7466	0
US1051323	WASHINGTON	TX	7482	7466	0
US1051323	WASHINGTON	UT	7482	7466	0
US1051323	WASHINGTON	VA	7482	7466	0
US1051323	WASHINGTON	VT	7482	7466	0
US1051323	WASHINGTON	WV	7482	7466	0

**Table 2:** This table describes the type of attributes we constructed for each potential location in step 2.

**Variable**	**Type**	**Comment**
Min. Location	Integer	Candidate Location (CL) first appearance in the document.
Street	Dummy	1 if CL is located to the right of the words ‘ST’, ‘AVENUE’, ‘ROAD’, ‘RD’, ‘BLVD’, or ‘AVE’. 0 otherwise.
City	Dummy	1 if ‘CITY’ is part of the CL name.
Frequency	Integer	Number of times the CL was found.
State	Dummy	1 if the name of the corresponding state was found. 0 otherwise
Min. Location State	Integer	State first appearance in the document.
State Distance	Set of dummy variables	Dummies corresponding to intervals of character distances between CL and the state name. The base category 0 includes also cases where the state name hasn’t been found.
Countries	Dummy	1 if the following words appear in the document: ‘GERMANY’, ‘ENGLAND’, ‘FRANCE’, ‘GREAT BRITAIN’, ‘SCOTLAND’, ‘IRELAND’, or ‘CANADA’. O otherwise.
Country Distance	Dummy	1 if a country name as specified above is found close to the CL. 0 otherwise.
Cutoff	Dummy	1 if the CL or the state has been found after the 50% of the document length. 0 otherwise.
Substring	Dummy	1 if the CL is a substring of another CL within the same patent. 0 otherwise. (i.e., York for New York).
Nchar	Integer	Number of characters of the CL.
Detected Name	Dummy	1 if the CL matches any part of the inventor or assignee name. 0 otherwise.
W State	Dummy	1 if at least 1 state name has been found for other CL within the same patent document. 0 otherwise.
Rel. Min. Location	Countinuous	Min Location over the length of the document. Varies between 0 and 1.
City	Dummy	1 if the CL was found next to the word ‘CITY’. 0 otherwise.
County	Dummy	1 if the CL was found next to the word ‘COUNTY’. 0 otherwise.
COC	Dummy	1 if more than one CL of the same county ID co-occur within the same patent document. 0 otherwise.
WX	Continuous	Index constructed with all the aforementioned variables for ‘competing’ CL within the same patent, as it is usual in spatial settings.

**Table 3:** This table only shows the locations that were predicted as correct by our statistical procedure.

**Publication Number**	**City**	**State**	**Length**	**Location**	**State Located**	**Y Hat**
US1051323	ELDORA	IA	7482	150	1	1
US1051323	HARDIN	IA	7482	426	1	0.99
In this example we set-up a minimal threshold of μ=0.5 for $\hat{Y}$, so we only classify as correct and keep those locations predicted as true with a likelihood above 50%.						

**Table 4:** This table shows an example of input for step 3, used to retrieve the location of unreadable patent documents.

**Inventor**	**PN**	**County**	**State**	**Year**	**Class**	**Frequency**	**Class Coinc.**	**Year Coinc.**
A. G. Bell	US1410875	DC	DC	1922	114	7	1	1
A. G. Bell	US1410875	Essex	MA	1922	114	2	0	0
A. G. Bell	US1410875	Middelsex	MA	1922	114	1	0	0
A. G. Bell	US1410875	Suffolk	MA	1922	114	3	0	0
A. G. Bell	US1410877	DC	DC	1922	114	7	1	1
A. G. Bell	US1410877	Essex	MA	1922	114	2	0	0
A. G. Bell	US1410877	Middelsex	MA	1922	114	1	0	0
A. G. Bell	US1410877	Suffolk	MA	1922	114	3	0	0
A. G. Bell	US181553	DC	DC	1876	310	7	0	0
A. G. Bell	US181553	Essex	MA	1876	310	2	0	4
A. G. Bell	US181553	Middelsex	MA	1876	310	1	0	0
A. G. Bell	US181553	Suffolk	MA	1876	310	3	0	0
A. G. Bell	US213090	DC	DC	1879	381	7	3	1
A. G. Bell	US213090	DC	DC	1879	379	7	5	1
A. G. Bell	US213090	Essex	MA	1879	379	2	1	0
A. G. Bell	US213090	Essex	MA	1879	381	2	0	0
A. G. Bell	US213090	Middelsex	MA	1879	381	1	0	0
A. G. Bell	US213090	Middelsex	MA	1879	379	1	0	0
A. G. Bell	US213090	Suffolk	MA	1879	379	3	2	0
A. G. Bell	US213090	Suffolk	MA	1879	381	3	4	0

**Table 5:** This table describes the type of attributes we constructed for each potential location in step 3.

**Variable**	**Type**	**Comment**
State	Dummy	1 if the name of the corresponding state was found. 0 otherwise.
Name Match	Dummy	1 if any place name within potential counties from stage 2 is found. 0 otherwise.
Nmatch	Integer	Number of name matches in ‘Name Match’.
Frequency PAL	Integer	Frequencies for locations obtained from the Patent to Assignee to Location Network (PAL) as described in Fig. 4.
Frequency PIL	Integer	Frequencies for locations obtained from the Patent to Inventor to Location Network (PIL) as described in Fig. 4.
Proportion PAL	Continuous	Proportion for locations obtained from the Patent to Assignee to Location Network (PAL) as described in Fig. 4.
Proportion PIL	Continuous	Proportion for locations obtained from the Patent to Inventor to Location Network (PIL) as described in Fig. 4.
Frequency PACL	Integer	Frequencies for locations obtained from the Patent to Assignee to Location to Class Network (PACL) as described in Fig. 4. It includes the restriction that the patent class should be the same to create a link between inventors name.
Frequency PICL	Integer	Frequencies for locations obtained from the Patent to Inventor to Location to Class Network (PICL) as described in Fig. 4. It includes the restriction that the patent class should be the same to create a link between inventors name.
Proportion PACL	Continuous	Proportion for locations obtained from the Patent to Assignee to Location to Class Network (PACL) as described in Fig. 4. It includes the restriction that the patent class should be the same to create a link between inventors name.
Proportion PICL	Continuous	Proportion for locations obtained from the Patent to Inventor to Location to Class Network (PICL) as described in Fig. 4. It includes the restriction that the patent class should be the same to create a link between inventors name.
Frequency PAYL	Integer	Frequencies for locations obtained from the Patent to Assignee to Location to Year Network (PAYL) as described in Fig. 4. It includes the restriction that the patent publication year should be between a span of 5 years to create a link between inventors name.
Frequency PIYL	Integer	Frequencies for locations obtained from the Patent to Inventor to Location to Year Network (PIYL) as described in Fig. 4. It includes the restriction that the patent publication year should be between a span of 5 years to create a link between inventors name.
Proportion PAYL	Continuous	Proportion for locations obtained from the Patent to Assignee to Location to Year Network (PAYL) as described in Fig. 4. It includes the restriction that the patent publication year should be between a span of 5 years to create a link between inventors name.
Proportion PIYL	Continuous	Proportion for locations obtained from the Patent to Inventor to Location to Year Network (PIYL) as described in Fig. 4. It includes the restriction that the patent publication year should be between a span of 5 years to create a link between inventors name.
Ubiquity	Integer	Ubiquity of the name of the inventor/assignee.
WX	Continuous	Index constructed with all the aforementioned variables for ‘competing’ CL within the same patent, as it is usual in spatial settings.
